# Optimized Loading of Idarubicin in CalliSpheres^®^ Drug-Eluting Beads and Characterization of Release Profiles and Morphological Properties

**DOI:** 10.3390/pharmaceutics13060799

**Published:** 2021-05-27

**Authors:** Enhao Lu, Guoliang Shao, Jingqin Ma, Yiwei He, Yuanchuan Gong, Zhiping Yan, Xianyi Sha

**Affiliations:** 1 Key Laboratory of Smart Drug Delivery, Department of Pharmaceutics, School of Pharmacy, Fudan University, Shanghai 201203, China; 19211030026@fudan.edu.cn; 2 Cancer Hospital of the University of Chinese Academy of Sciences (Zhejiang Cancer Hospital), Hangzhou 310022, China; shaogl@zjcc.org.cn (G.S.); 202011122211490@zcmu.edu.cn (Y.H.); 20021051062@usx.edu.cn (Y.G.); 3 Institute of Cancer and Basic Medicine (IBMC), Chinese Academy of Sciences, Hangzhou 310000, China; 4 Interventional Radiology of Zhongshan Hospital, Fudan University, Shanghai 200032, China; ma.jingqin@zs-hospital.sh.cn

**Keywords:** CalliSpheres^®^, idarubicin, loading efficiency, release profiles, morphology, iopamidol compatibility

## Abstract

This study aimed to investigate the idarubicin loading method, compatible stability with contrast agent, release profiles, and morphological properties of 50–150, 100–300, and 300–500 μm CalliSpheres^®^. The amounts of idarubicin added, loading medium, loading condition, and drug concentration were investigated as factors influencing drug loading efficiency. The drug loading rate was negatively correlated with the amount drug added and diameter of CalliSpheres^®^ and positively correlated with the drug concentration. Compared to loading in purified water and incubation at room temperature, 5% glucose, heating, and ultrasound could accelerate drug loading. The idarubicin loading efficiency was above 95% after 10 min for all three CalliSpheres^®^ with the optimized method of adding 20 mg of idarubicin at a concentration of 2 mg/mL and incubating at room temperature. The drug leak rate was under 1% within 8 h after mixing with iopamidol. Drug release tests indicated the sustained-release performance of CalliSpheres^®^, and the time to reach 75% of the release plateau level was 8, 26, and 51 min for 50–150, 100–300, and 300–500 μm CalliSpheres^®^, respectively. After idarubicin loading, the diameters increased by 12%, 36%, and 38% for 50–150, 100–300, and 300–500 μm CalliSpheres^®^, respectively, and the surface of CalliSpheres^®^ was observed to become smoother than that before drug loading. All three CalliSpheres^®^ presented satisfactory loading efficiency with the optimized method, as well as proper compatible stability and sustained release performance. Among them, 100–300 μm CalliSpheres^®^ are recommended.

## 1. Introduction

Transarterial chemoembolization (TACE) is the most commonly used therapy in unresectable hepatocellular carcinoma (HCC) [1]. Compared to iodipin or other embolic particles, drug-eluting embolic bead (DEB) is widely accepted for its long-term embolic effect and sustained drug release [2]. Idarubicin (IDA) is a derivative of doxorubicin, which has better lipophilicity, meaning its uptake through the cellular membrane is improved [3]. The application of IDA in TACE has been proved by previous studies [4,5], which have also revealed some advantages that IDA might have in HCC therapy [6,7].

Some previous studies have investigated the ability to load IDA to classic embolic beads [8], such as DC Bead^TM^ (BTG, Surry, UK) and HepaSphere^TM^ (Merit Medical, South Jordan, UT, USA). However, the detailed IDA loading profiles, such as dosage, concentration, and drug loading time and medium, have not been studied clearly. These features are key issues for clinical application, since unloaded IDA mixed in DEB might cause a rapid release to the systemic circulation [9]. In addition, the compatible stability with contrast agent and surface morphology of DEB after drug loading have not been investigated. Further investigations of the characteristics of DEB as IDA carriers are needed, as well as the comparison of DEB with other types of porous delivery carriers [10].

CalliSpheres^®^ (Callisyn Biomedical-Suzhou, Inc., Suzhou, China) is a novel DEB, which was approved for clinical use in 2015 in China and obtained FDA 510 (k) certification in 2018 [11]. CalliSpheres^®^ have a similar chemical structure and application property to DC Bead, but at a lower price [12]. Previous studies have investigated the drug loading of some classic antitumor agents to CalliSpheres^®^, such as doxorubicin [13], oxaliplatin [14], irinotecan [15], and vinorelbine [16]. However, the drug delivery ability of IDA has not yet been reported.

In this present study, detailed IDA loading features and optimized loading methods were investigated for three CalliSpheres^®^ of different particle size, as well as their compatible stability, release profiles, and morphological changes. The authors aimed to provide solutions for clinical application by this in vitro study.

## 2. Materials and Methods

### 2.1. Materials

CalliSpheres^®^ (Callisyn Biomedical-Suzhou, Inc., Suzhou, China) with sizes of 50–150, 100–300, and 300–500 μm were evaluated. Each package contains 1 g of DEB and 7 of mL normal saline, making up a total volume of about 8 mL. Idarubicin hydrochloride for injection (National Medicine Permission Number H20203345, Batch No. 200917) and iopamidol injection (National Medicine Permission Number H20203293, Batch No. 200812) were kindly provided by Chia Tai Tianqing Pharmaceutical Group Co. Ltd., Nanjing, China. Each IDA package contains hydrochloride powder with 10 mg of active pharmaceutical ingredients (APIs). Idarubicin hydrochloride standard (>99%) was purchased from the National Institutes for Food and Drug Control, China. Methyl alcohol (HPLC grade) was purchased from Sigma-Aldrich, Saint Louis, MO, USA. Phosphate buffered saline (1 × PBS, pH = 7.2~7.4) and fetal bovine serum (FBS) were purchased from Gibco, Grand Island, NY, USA. The 0.9% sodium chloride for injection (0.9% NaCl) and the 5% glucose for injection (5% Glu) were purchased from Shijiazhuang No. 4 Pharmaceutical, China. Ultrapure water (H_2_O) was obtained from a Millipore system (Millipore, Bedford, MA, USA). All other chemicals were of analytical grade.

### 2.2. Screening of IDA Loading Method

The amount IDA added, loading medium, loading condition, and IDA concentration were investigated. CalliSpheres^®^ with different particle sizes of 50–150, 100–300, and 300–500 μm were evaluated in this part. The drug solution was prepared by adding H_2_O, 0.9% NaCl, or 5% Glu to a package of idarubicin hydrochloride for injection to produce the required concentrations. CalliSpheres^®^ were rinsed by the corresponding medium three times, discarding the liquid supernatant and setting it aside. IDA solution was then added to CalliSpheres^®^. After gentle agitation, the mixture was incubated at room temperature (37 °C) or in a running ultrasound cleaner (SK2510HP, Shkedao, Shanghai, China). Liquid supernatant was collected at 0, 5, 10, 15, 30, 45, 60, and 90 min, diluted by mobile phase-methyl alcohol and 0.1% phosphoric acid at a ratio of 70:30 (*v/v*), and tested by an Agilent 1260 high-performance liquid chromatography (HPLC) system (Agilent Technologies Co., Santa Clara, CA, USA), equipped with an Agilent G4212B DAD detector and an Agilent Eclipse Plus C18 column (4.6 × 150 mm, 5 μm). The mobile phase was methyl alcohol–0.1% phosphoric acid (*v*/*v*, 70:30), at a rate of 1.0 mL/min. Other parameters are as follows: column temperature = 25 °C, injection volume = 10 μL, UV wavelength = 254 nm, and running time = 10 min. The efficiency of loading was calculated as follows:

$$\mathrm{Loading}\;\mathrm{efficiency}\left(\%\right)=\frac{\mathrm{initial}\;\mathrm{IDA}\;\mathrm{concentration}\;-\;\mathrm{residual}\;\mathrm{IDA}\;\mathrm{concentration}\;\mathrm{in}\;\mathrm{supernatant}}{\mathrm{initial}\;\mathrm{IDA}\;\mathrm{concentration}}\times 100\%$$

In the screening of the amount of IDA to add, the experimental groups were set as 5, 10, 15, 20, and 40 mg, for each marketed CalliSpheres^®^ preparation, while other factors were fixed as follows: loading medium = purified H_2_O, loading condition = room temperature, and drug concentration = 500 μg/mL. In the screening of loading medium, the experimental groups were set as purified H_2_O, 0.9% NaCl, and 5% Glu, while the other factors were fixed as follows: amount of IDA to add = 20 mg, loading condition = room temperature, and drug concentration = 500 μg/mL. In the screening of the loading condition, the experimental groups were set as incubation at room temperature (37 °C) and in an ultrasound cleaner, while the other factors were fixed as follows: amount of IDA to add = 20 mg, loading medium = purified H_2_O, and drug concentration = 500 μg/mL. In the screening of IDA concentration, the experiment groups were set as 0.25, 0.5, 1, 1.5, and 2 mg/mL, while the other factors were fixed as follows: amount of IDA to add = 20 mg, loading medium = purified H_2_O, and loading condition = room temperature.

### 2.3. Stability after Compatibility with Contrast Agent

IDA was loaded by the optimized loading method to 100–300 and 300–500 μm CalliSpheres^®^, at an amount of 20 mg for each marketed CalliSpheres^®^ preparation. A 10 mL iopadol injection, equivalent to 3.7 g iopadol, was added, and, after gentle agitation, the mixture was incubated at room temperature. The IDA concentration in the mixed liquid was tested at 0, 10, 20, 30, and 45 min and then at 1, 1.5, 2, 4, and 8 h, by the above-mentioned HPLC method. The IDA leak rate was calculated as follows:

$$\mathrm{Leak}\;\mathrm{rate}\left(\%\right)=\frac{\;\mathrm{IDA}\;\mathrm{concentration}\;\mathrm{at}\;\mathrm{sample}\;\mathrm{time}\times \mathrm{volume}}{\mathrm{IDA}\;\mathrm{adding}\;\mathrm{amount}-\mathrm{IDA}\;\mathrm{concentration}\;\mathrm{at}\;0\;\min\times \mathrm{volume}}\times 100\%$$

### 2.4. Assessment of IDA Release Profiles

The IDA release rate was measured by a Pharmacopeia flowthrough apparatus 4 (USP 4) Sotax system (CE 7smart, SOTAX AG, Aesch, Switzerland). For each size of CalliSpheres^®^, the drug release rate was tested in PBS and PBS + 10% FBS separately to investigate the influence of FBS. The other parameters were as follows: bath temperature = 37 °C, flow rate = 5.0 mL/min, and medium volume = 200 mL. IDA concentration was evaluated at 10, 20, 30, and 45 min and then at 1, 1.5, 2, 3, 4, 6, 8, 12, 24, 48, and 72 h. Before analysis by HPLC, each solution sample was mixed with methyl alcohol at a volume ratio of 1:1, followed by high-speed centrifugation (10,000× *g*, 5 min), for protein precipitation. In addition, free drug of IDA was also evaluated as a control group, in order to correct the drug degradation during the experiment.

### 2.5. Morphological and Physical Properties

Morphological changes of CalliSpheres^®^ before and after IDA loading were detected by a scanning electron microscope (SEM, Nova NanoSEM 450, FEI, Hillsboro, OR, USA). The sample preparation process was as follows: 50 μL of DEB suspension were first spread on cover slips, dried overnight at room temperature, and metal was sprayed for 2 min by an ion sputtering machine (SBC-12, KYKY, Beijing, China). The SEM parameters were as follows: condition = high vacuum, mode = secondary electrons with E-T detector, and accelerating voltage = 5 kV. Changes in color and size of CalliSpheres^®^ were detected by an inverted fluorescence microscope (DMI4000 B, Leica, Solms, Germany). Quantitative particle sizes were measured by a laser diffraction particle size analyzer (Mastersizer 3000, Malvern, UK).

### 2.6. Statistics

Data are mainly presented as mean ± standard deviation. Statistical analyses and plotting were performed using SPSS Statistics 23 and GraphPad Prism 8. Comparisons among groups were determined by t-tests or analysis of variance. *p* < 0.05 was considered significant.

## 3. Results

### 3.1. IDA Loading Method

**Figure 1:**
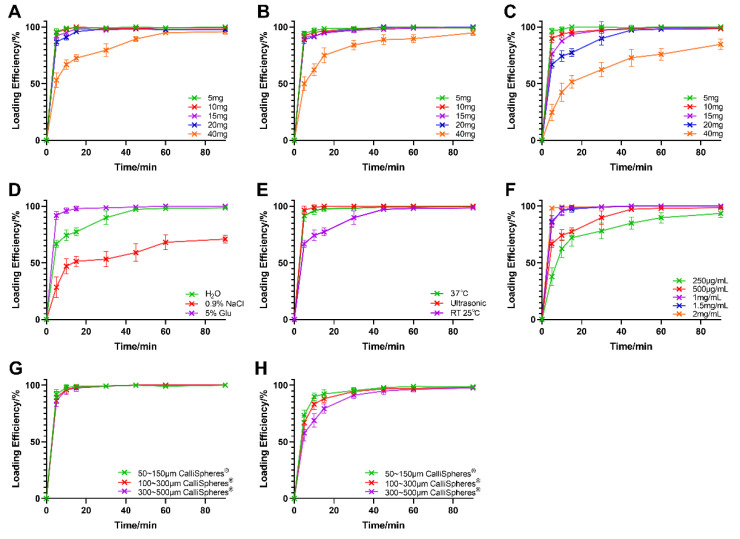
IDA loading efficiency with different loading methods (*n* = 3). The results of screening the amount of drug to add in three CalliSpheres^®^ of different particle sizes: (**A**) 50–150 μm; (**B**) 100–300 μm; and (**C**) 300–500 μm. The results of further loading method screening: (**D**) loading medium; (**E**) loading condition; and (**F**) drug concentration. The IDA loading curves by the optimized loading method are shown (**G**). The results of ultimate loading test are shown (**H**).

As presented in Figure 1A–C, for an IDA concentration of 500 μg/mL, the loading efficiency was above 95% for all three CalliSpheres^®^, while the added amount was under 20 mg, whereas the drug-loading speed decreased with an increase in particle sizes. When the amount of IDA added increased to 40 mg, however, only 50–150 μm CalliSpheres^®^ enabled a loading efficiency of 90% at 1 h after the beginning of drug loading. These results imply that the IDA loading ability for CalliSpheres^®^ was lower for larger sizes than for smaller ones.

According to clinical application, 20 mg of IDA are adequate for a single embolization treatment [7]. However, considering the convenience of clinical use, a higher IDA loading speed—for example, above 90% in 15 min—is needed. In the screening of IDA amount to add, 300–500 μm CalliSpheres^®^ showed poor drug loading efficiency in the objective drug loading amount (20 mg), while the loading efficiency was already over 95% within 15 min in 50–150 and 100–300 μm CalliSpheres^®^. Thus, the influences of the loading medium, loading condition, and drug concentration were further investigated based on 300–500 μm CalliSpheres^®^. As shown in Figure 1D, 5% Glu was beneficial for drug loading, while 0.9% NaCl had the opposite effect. As for the loading condition, it was revealed that ultrasound and heating could accelerate drug loading (Figure 1E). Regarding the drug concentration, IDA loading efficiency increased when the concentration increased from 250 to 2 mg/mL, which indicated that higher drug concentration promoted drug loading.

For convenience, the strategy of increasing the IDA concentration was selected. The optimized IDA loading method is summarized as follows: amount of IDA to add = 20 mg, IDA concentration = 2 mg/mL, medium = purified water, and loading condition = incubation at room temperature. Drug loading curves for the optimized method are shown in Figure 1G. The loading efficiency was above 95% at 10 min for all three CalliSpheres^®^. An ultimate loading test was also conducted. Upon increasing the IDA amount to 40 mg with all other parameters being the same, the drug loading efficiency exceeded 90% at 30 min (Figure 1H).

### 3.2. Stability after Compatibility with Contrast Agent

**Figure 2:**
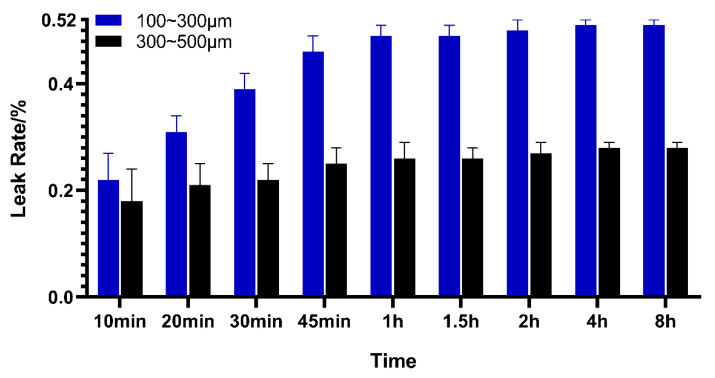
IDA leak rate after compatibility with iopamidol (*n* = 3).

After adding iopamidol to CalliSpheres^®^, it was observed that the drug-eluting beads were suspended in the liquid, which might have caused the increased density of the mixed solution. The IDA leak rate is presented in Figure 2. Drug leaks reached equilibrium at about 1 h after adding contrast agent. The stabilized leak rates were 0.51% ± 0.01% for 100–300 μm CalliSpheres^®^ and 0.28% ± 0.01% for 300–500 μm CalliSpheres^®^.

### 3.3. IDA Release Profiles

**Figure 3:**
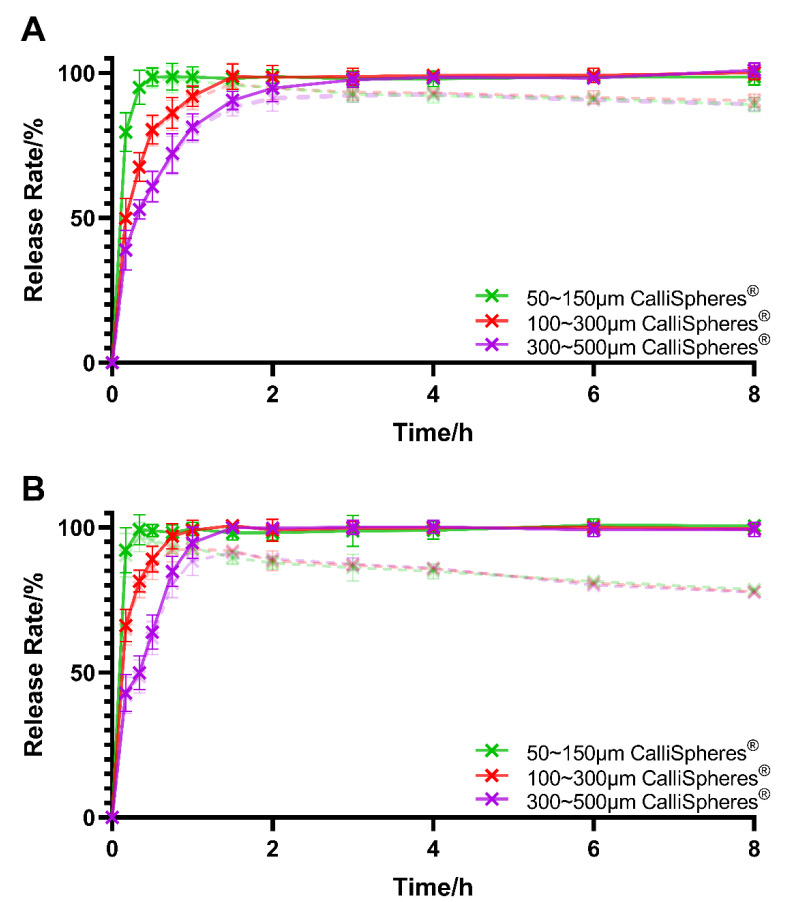
IDA in vitro release profiles in: (**A**) PBS; and (**B**) PBS + 10% FBS. The dashed lines are the release rate before correction of drug degradation (*n* = 3).

The release profiles of CalliSpheres^®^ with 20 mg of IDA loaded in PBS and PBS + 10% FBS are shown in Figure 3. The IDA concentration in the control group decreased during the experiment, which was consistent with previous reports [8]. The results reveal that CalliSpheres^®^ had a sustained release ability, and the release rate decreased with an increase in the CalliSpheres^®^ particle sizes. In addition, FBS significantly accelerated the drug release in the first 1.5 h (*p* < 0.05).

### 3.4. Morphological and Physical Properties

The results of particle size measurement are shown in Table 1, which indicated that the diameters of CalliSpheres^®^ increased after being rinsed by H_2_O and later decreased after loading IDA. From the initial marketed products to the final IDA-loaded DEB, there was a statistically significant diameter increase in all three CalliSpheres^®^.

**Figure 4:**
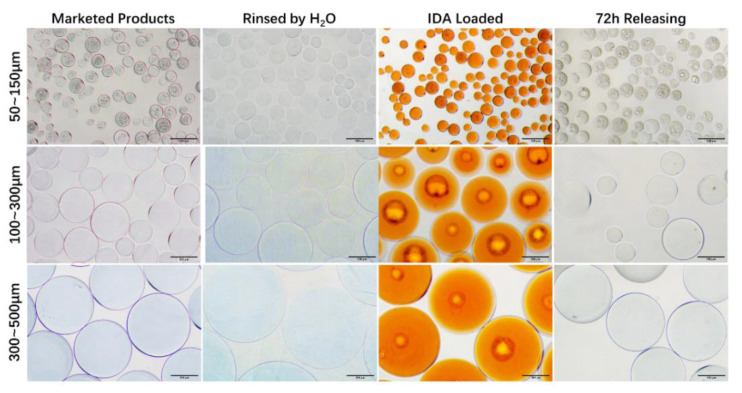
Optical microscope images of CalliSpheres^®^ before/after IDA loading and release. Scale bars: 200 μm.

The morphology before/after drug loading and release under an optical microscope is shown in Figure 4. The color of CalliSpheres^®^ turned from blue to orange after IDA loading and faded after the drug release. The black circular stripe is the contact surface of CalliSpheres^®^ and glass slide, under the inverted microscope. The surface of 50–150 μm CalliSpheres^®^ was bumpier than the larger ones, which might be ascribed to the preparation technology of CalliSpheres^®^ of different sizes.

**Figure 5:**
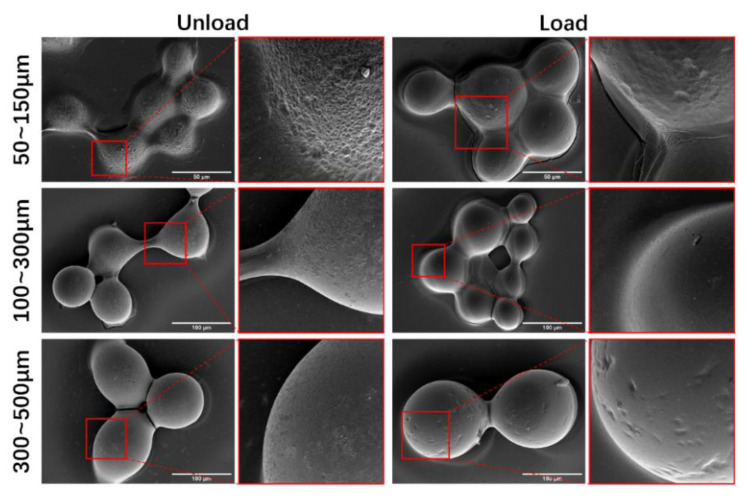
SEM images of CalliSpheres^®^ before/after IDA loading. Scale bars in main the pictures: 50 μm for 50~150 μm CalliSpheres^®^, and 100 μm for 100~300 μm and 300~500 μm CalliSpheres^®^.

SEM disclosed that CalliSpheres^®^ presented small holes before IDA loading, whereas these holes became smooth after drug loading (Figure 5).

## 4. Discussion

Similar to that of doxorubicin, the drug-loading mechanism of IDA is an electrostatic interaction, based on the negative charges of sulfonic acid groups in CalliSpheres^®^ and the positive charges of primary amino groups in IDA. In the screening test for the IDA loading method, 0.9% NaCl was shown to adversely affect to drug loading, which is probably because the ion effects in NaCl solution weakened the electrostatic attraction between CalliSpheres^®^ and IDA. On the contrary, 5% Glu solution is slightly acidic, which promoted the protonation of amino groups in IDA, thus accelerating the drug-loading process. Compared to simple incubation at room temperature, ultrasound and heating enhanced the frequency of contact between IDA and CalliSpheres^®^, thereby promoting drug loading. In general, compared to ultrasound or heating, increasing the IDA concentration is a more convenient strategy with which to accelerate drug loading in clinical settings. Therefore, incubation at room temperature and 2 mg/mL IDA concentration were finally selected.

In clinical applications, DEB is often administered in combination with a contrast agent, such as iopamidol or iopromide [1]. Compatible stability is thus an important issue in clinical medication practice but rarely investigated in previous studies. In consideration of the drug-loading mechanism, adding a contrast agent to DEB might lead to drug leakage. According to the present research, however, the drug leak rate was proved to be under 1% within 8 h after mixing with iopamidol, showing this IDA delivery system to have compatible stability.

The selection of particle sizes for DEB is another important issue in clinical practice [9]. It is widely believed that embolic agents in small scale will embolize vascular endings, which enhances the curative effect. Particles that are too small, however, create the potential for incomplete embolization or even ectopic embolism. In terms of DEB, the diameter of microspheres will also influence the loading and release profiles. In this present study, 50–150 μm CalliSpheres^®^ had higher drug-loading speed, while 300–500 μm ones showed great advantages in terms of sustained drug release. These results make sense because smaller CalliSpheres^®^ have larger specific surface areas, which accelerate drug loading and release. Considering both the embolism effect and sustained-release performance, 100–300 μm CalliSpheres^®^ are recommended. Compared to previous studies on other classic marketed DEB, the release plateau level of CalliSpheres^®^ could reach 100%, which was much higher than those for DC Bead^TM^ (74% ± 3%), HepaSphere^TM^ (65% ± 6%), or LifePearl^TM^ (73% ± 3%) [8]. As for the elution rate, the time to reach 75% of the release plateau level was 8 min for 50–150 μm CalliSpheres^®^, 26 min for 100–300 μm CalliSpheres^®^, and 51 min for 300–500 μm CalliSpheres^®^. Compared to DC Bead^TM^ with 100–300 μm particle sizes, which takes 24 min to reach 75% of the release plateau level (5 mg IDA loaded) [8] or 37 min (10 mg IDA loaded) [5], the eluting rate of CalliSpheres^®^ is higher. In addition, it was indicated that FBS would accelerate IDA release, probably because some components in FBS weakened the electrostatic interaction.

Regarding the physical properties of CalliSpheres^®^, there was a variation in the trend of diameters before/after rinsing and IDA loading, which was measured by laser diffraction particle size analyzer, and was consistent with the images produced by an optical microscope. In general, the diameters after IDA loading were larger than those of the original marketed products. Especially for 300–500 μm CalliSpheres^®^, the mean diameter after IDA loading was over 500 μm. These beads are unable to enter the branch blood vessels supplying a tumor and embolize them, thus 300–500 μm CalliSpheres^®^ are not recommended. IDA has an orange-red color, thus the general conditions of drug loading and release could be observed through the color of CalliSpheres^®^ seen under optical microscope. In SEM images, no drug crystals were observed on the surface of CalliSpheres^®^, which was different from the cases for some rapid-release drugs such as oxaliplatin [14]. These results indicate that IDA could distribute inside the beads, which also explained the sustained IDA release.

This study optimized the IDA loading method and evaluated the compatible stability, drug release profiles, and physical properties of CalliSpheres^®^ of three different particle sizes. To better simulate clinical practice, marketed idarubicin hydrochloride for injection was used in this research, as well as APIs. Limited by experiment conditions, however, no other IDA preparations from different manufacturers were evaluated. In addition, although the IDA release rate was tested by the USP 4 method, which is considered to have proper in vivo–in vitro correlation, the results cannot completely reflect the drug eluting process in TACE treatment of HCC.

## 5. Conclusions

This in vitro study investigated the IDA loading method, release profiles, and morphologic properties of CalliSpheres^®^. All three CalliSpheres^®^ of different particle sizes disclosed satisfactory loading efficiency with the optimized method, as well as sustained release performance in vitro. The optimized IDA loading method is of important significance for clinical applications, especially indicating that normal saline might not be an appropriate drug-loading medium. In addition, IDA-loaded CalliSpheres^®^ present great compatible stability with iopamidol within 8 h, meaning that pre-operation drug leaks do not need to be considered in most clinical applications. According to the experiment results and general clinical settings, 100–300 μm CalliSpheres^®^ are recommended.

## Figures and Tables

**Table 1:** Diameter changes in IDA loading process (*n* = 3).

	50–150 μm CalliSpheres^®^	100–300 μm CalliSpheres^®^	300–500 μm CalliSpheres^®^
Initial sizes (μm)	87.62 ± 16.05	212.95 ± 36.22	395.33 ± 36.46
After rinsing (μm)	131.59 ± 24.68	337.47 ± 34.93	618.13 ± 53.76
After IDA loading (μm)	98.25 ± 15.33	290.20 ± 35.76	544.87 ± 32.75
Expansion in diameter (%) (Compared with initial sizes)	12.13%, *p* = 0.038	36.28%, *p* < 0.01	37.83%, *p* < 0.01

## Data Availability

The study did not report any data.

## Abbreviations

TACE, transarterial chemoembolization; HCC, hepatocellular carcinoma; DEB, drug-eluting embolic bead; IDA, idarubicin; HPLC, high-performance liquid chromatography; PBS, phosphate buffered saline; FBS, fetal bovine serum; SEM, scanning electron microscope; APIs, active pharmaceutical ingredients.
